# A probabilistic cell model in background corrected image sequences for single cell analysis

**DOI:** 10.1186/1475-925X-9-57

**Published:** 2010-10-06

**Authors:** Nezamoddin N Kachouie, Paul Fieguth, Eric Jervis

**Affiliations:** 1 Department of Systems Design Engineering, University of Waterloo, Waterloo, Canada; 2 Department of Chemical Engineering, University of Waterloo, Waterloo, Canada; 3 Harvard-MIT Health Sciences and Technology Harvard Medical School, Cambridge, USA

## Abstract

**Background:**

Methods of manual cell localization and outlining are so onerous that automated tracking methods would seem mandatory for handling huge image sequences, nevertheless manual tracking is, astonishingly, still widely practiced in areas such as cell biology which are outside the influence of most image processing research. The goal of our research is to address this gap by developing automated methods of cell tracking, localization, and segmentation. Since even an optimal frame-to-frame association method cannot compensate and recover from poor detection, it is clear that the quality of cell tracking depends on the quality of cell detection within each frame.

**Methods:**

Cell detection performs poorly where the background is not uniform and includes temporal illumination variations, spatial non-uniformities, and stationary objects such as well boundaries (which confine the cells under study). To improve cell detection, the signal to noise ratio of the input image can be increased via accurate background estimation. In this paper we investigate background estimation, for the purpose of cell detection. We propose a cell model and a method for background estimation, driven by the proposed cell model, such that well structure can be identified, and explicitly rejected, when estimating the background.

**Results:**

The resulting background-removed images have fewer artifacts and allow cells to be localized and detected more reliably. The experimental results generated by applying the proposed method to different Hematopoietic Stem Cell (HSC) image sequences are quite promising.

**Conclusion:**

The understanding of cell behavior relies on precise information about the temporal dynamics and spatial distribution of cells. Such information may play a key role in disease research and regenerative medicine, so automated methods for observation and measurement of cells from microscopic images are in high demand. The proposed method in this paper is capable of localizing single cells in microwells and can be adapted for the other cell types that may not have circular shape. This method can be potentially used for single cell analysis to study the temporal dynamics of cells.

## Introduction

The automated acquisition of huge numbers of digital images has been made possible due to advances in and the low cost of digital imaging. In many video analysis applications, the goal is the tracking of one or more moving objects over time such as human tracking, traffic control, medical and biological imaging, living cell tracking, forensic imaging, and security [1-7].

The possibility of image acquisition and storage has opened new research directions in cell biology, tracking cell behaviour, growth, and stem cell differentiation. The key impediment on the data processing side is that manual methods are, astonishingly, still widely practiced in areas such as cell biology which are outside the influence of most image processing research. The goal of our research, in general, is to address this gap by developing automated methods of cell tracking.

Although most televised video involves frequent scene cuts and camera motion, a great deal of imaging, such as medical and biological imaging, is based on a fixed camera which yields a static background and a dynamic foreground. Moreover, in most tracking problems it is the dynamic foreground that is of interest, hence an accurate estimation of the background is desired which, once removed, ideally leaves us with the foreground on a plain background. The estimated background may be composed of one or more of random noise, temporal illumination variations, spatial distortions caused by CCD camera pixel non-uniformities, and stationary or quasi-stationary background structures.

We are interested in the localization, tracking, and segmentation of Hematopoietic Stem Cells (HSCs) in culture to analyze stem-cell behavior and infer cell features. In our previous work we addressed cell detection/localization [8,9] and the association of detected cells [10]. In this paper cell detection and background estimation will be studied, with an interest in their mutual inter-relationship, so that by improving the performance of the background estimation we can improve the performance of the cell detection. The proposed approach contains a cell model and a point-wise background estimation algorithm for cell detection. We show that point-wise background estimation can improve cell detection.

There are different methods for background modelling, each of which employs a different method to estimate the background based on the application at hand, specifies relevant constraints to the problem, and makes different assumptions about the image features at each pixel, processing pixel values spatially, temporally, or spatio-temporally [11-23].

There is a broad range of biomedical applications of background estimation, each of which introducing a different method to estimate the background based on some specific assumptions relevant to the problem [12-14,24]. Close and Whiting [12] introduced a technique for motion compensation in coronary angiogram images to distinguish the arteries and background contributions to the intensity. They modelled the image in a region of interest as the sum of two independently moving layers, one consisting of the background structure and the other consisting of the arteries. The density of each layer varies only by rigid translation from frame to frame and the sum of two densities is equal to the image density.

Boutenko *et. al *[13] assumed that the structures of interest are darker than the surrounding immobile background and used a velocity based segmentation to discriminate vessels and background in X-ray cardio-angiography images, considering the faster vessel motion in comparison with the background motion.

Chen *et. al *[14] modelled the background of a given region of interest using the temporal dynamics of its pixels in quantitative fluorescence imaging of bulk stained tissue. They modelled the intensity dynamics of individual pixels of a region of interest and derived a statistical algorithm to minimize background and noise to decompose the fluorescent intensity of each pixel to background and the stained tissue contributions.

A simulation and analysis framework to study membrane trafficking in fluorescence video microscopy was proposed by Boulanger *et. al *[24]. They designed time-varying background models in fluorescence images and proposed statistical methods for estimating the model parameters. This method decides whether any image point belongs to the image background or a moving object.

Several segmentation and tracking methods are proposed for a broad range of biomedical applications, each of which introducing a different method to segment and/or track specific biological materials based on some specific assumptions relevant to the problem [25-28].

Cheng *et. al *used shape markers to separate clustered nuclei from fluorescence microscopy cellular images in a watershed-like algorithm [25]. Shape markers were extracted using H-minima transform. A marking function was introduced to separate clustered nuclei while geometric active contour was used for initial segmentation.

Gudla *et. al *proposed a region growing method for segmentation of clustered and isolated nuclei in fluorescence images [26]. They used a wavelet-based approach and a multi-scale entropy-based thresholding for contrast enhancement. They first oversegmented nuclei and then merged the neighboring regions into single nuclei or clustered nuclei based on area followed by automatic multistage classification.

A semi-automatic mean-shift-based method for tracking of migrating cell trajectories *in vitro* phase-contrast video microscopy was proposed by Debeir *et. al *[28]. They used mean-shift principles and adaptive combinations of linked kernels in the proposed method. They used this method for detection of different gray-level configurations. This method required manual initialization of the cell centroids on the first frame, it did not use temporal filtering or time-dependent feature, and it did not provide precise information on the cell boundaries and shapes.

Most tracking problems have an implicit, nonparametric model of the background to avoid making assumptions regarding the foreground. By developing a model for the background it is possible to find a classifier that labels each image pixel as background/not background; i.e., the foreground is identified as that which is not background. In contrast, the more focussed context of our cell tracking problem admits an explicit model of the foreground. Because of the low SNR of our problem, where illumination is limited to minimize cell phototoxicity, it is desired to remove all deterministic non-cell variations in the image (i.e., the background) before localizing the cells.

Some of the earlier works have integrated foreground detection and background estimation in a mutual framework, however most of the previous methods classify each pixel to either foreground or background, where their goal is the general segmentation of dynamic objects with no assumptions regarding the foreground. In contrast our goal is the localization of foreground objects, given specific assumptions that are integrated in the form of a foreground model.

In our proposed method, in place of classifying each pixel to either foreground or background, we estimate a single global background and do detection of foreground objects (but not pixel by pixel). Our proposed method addresses foreground detection and background estimation as inter-related processes, and take advantage of this inter-relation to improve the performance of cell detection. In the proposed algorithm, the background elements are removed from the scene frame by frame using a spatio-temporal background estimator while a probabilistic cell model is applied to the image sequence to localize cell centers. The spatio-temporal estimator has been applied to estimate the background in phase contrast image sequences taken from living Hematopoietic (blood) Stem Cells in culture, and leads to substantial improvements in cell localization and cell outline detection.

## Materials

To produce the data for this study, HSC samples are first extracted from mouse bone marrow, then cultured in custom arrays of microwells. The cells were imaged using manual focusing through a 5× phase contrast objective using a digital camera (Sony XCD-900) and acquired by an IEEE 1394 standard (FireWire) connector. Images were sampled every three minutes over the course of several days. During imaging, chambers were maintained at 37°C, in a 5% CO_2 _humidified air environment.

## Methods

**Figure 1:**
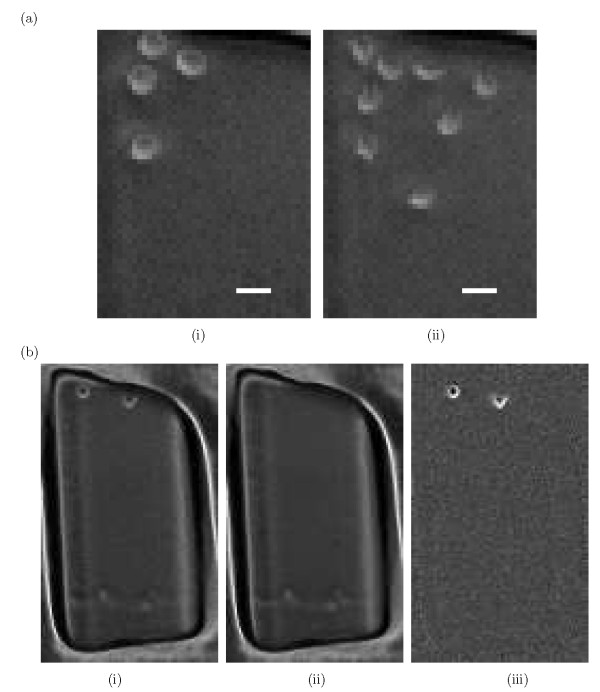
**Background estimation**. (a) Two unprocessed blood stem cell images: (i) Frame 1. (ii) Frame 50. Scale bar is 20 *μm*. (b) Coarse cropped well: (i) Coarsely cropped well in which well boundaries are visible. (ii) Estimated background obtained by applying the point-wise method ${\hat{B}}^{0}$. (iii) Panel (i) after background subtraction.

Two original frames taken from a cropped well is depicted in Fig. 1(a-i) and 1(a-ii). Well cropping is often approximate and the well boundaries may be partially or completely visible in the cropped image sequence, as can be seen in Fig. 1(b-i). Modelling cells on a uniform, zero-mean background requires that any existing background be estimated and subtracted.

A probabilistic cell model is proposed as the product of two probabilistic terms associated with the cell brightness and the surrounding background. We also propose a background estimation method which is driven by the proposed cell model to identify and reject the well structures. In this section the background estimation method is first presented followed by the proposed probabilistic cell model.

### Point-wise Background Estimation for Cell Detection

To estimate the background, let image *I*_*k *_be defined on a fixed lattice *L*:

(1) $$I_{k}=\{I_{ijk}|(i,j)\in L\}$$

thus for each frame *I*_*k *_of an image sequence we can write

(2) $$I_{k}=F_{k}+B+n_{k}\cdot 1+V_{k}$$

where *F*_*k *_is the dynamic foreground, *B *is the fixed background, *n*_*k *_models the temporal variations in global lighting, and *V*_*k *_is spatio-temporal random additive noise. The temporal noise *n*_*k *_is estimated over all pixels in each frame *k*

(3) $${\hat{n}}_{k}=\underset{\left\{ij\right\}}{mean}\;\;\;\;(I_{ijk})\;\;\;\;\;\;\{(i,j)\in L\}$$

For temporal correction, the estimated temporal noise is subtracted from the original frame *I*_*k*_:

(4) $$g_{k}=I_{k}-{\hat{n}}_{k}\cdot 1$$

The true background *B *= [*B*_*ij*_] is composed of stationary distortions and illumination variations at each pixel location. The point-wise estimation ${\hat{B}}^{0}$of the background can be estimated over *K *frames of temporal corrected sequence **g **= (g_1_, g_2_,..., g_K_):

(5) $${\hat{B}}_{ij}^{0}=\underset{\left\{k\in [1,K]\right\}}{mode}\;\;\;\;\;\;(g_{\left(i,j,k\right)})$$

and is subtracted out from **g**:

(6) $$\hat{F}=\text{g}-{\hat{B}}^{0}$$

An imperfectly cropped well, the corresponding estimated background, and the corrected image are depicted in Figs. 1(b-i), (b-ii), and 1(b-iii) respectively.

#### Justification of mode as statistical measure

Empirically, the motion of blood stem cells is essentially random, especially when observed minutes apart. Since cell motion is rarely zero, the spatial variations in cell brightness, mean that the variability of an image pixel, located within a cell, is considerably higher than the variability of an image pixel lieing in the background, whose variability is due only to random noise. Therefore, excepting cases of unusually small cell motion, the distribution of brightness values at a pixel should be most sharply peaked at the background, which is therefore recovered by the mode of the sample histogram.

### Cell Detection in a Uniform Background

Let **I **= (*I*_1_, *I*_2_,..., *I*_*K *_) be a set of *K *images which we will assume to consist of cells on a plain, uniform background. A typical microscopic multi-well image sequence **I **in our experiments consists of 32 separated wells, in each of which two to four HSCs are injected. Single-well image sequences are cropped from the original multi-well image sequence and are processed individually. Frames 1 and 50 of a typical cropped well are depicted in Fig. 1(a).

In our previously designed [8] cell detection method, stem-cell center locations were inferred from an image *I*_*k *_by

(7) Pold(zkm|Ik)=Pcb(zkm|Ik)⋅Pin(zkm|Ik)⋅Pcdf(zkm|Ik)

where $z_{k}^{m}=(x_{k}^{m},y_{k}^{m},r_{k}^{m})$ represents a cell with radius $r_{k}^{m}$ located at coordinate $\left(x_{k}^{m},y_{k}^{m}\right)$, and where *P*_*cb*_, *P*_*in *_and *P*_*cdf *_characterize the bright cell boundary, the dark cell interior, and the boundary uniformity, respectively.

**Figure 2:**
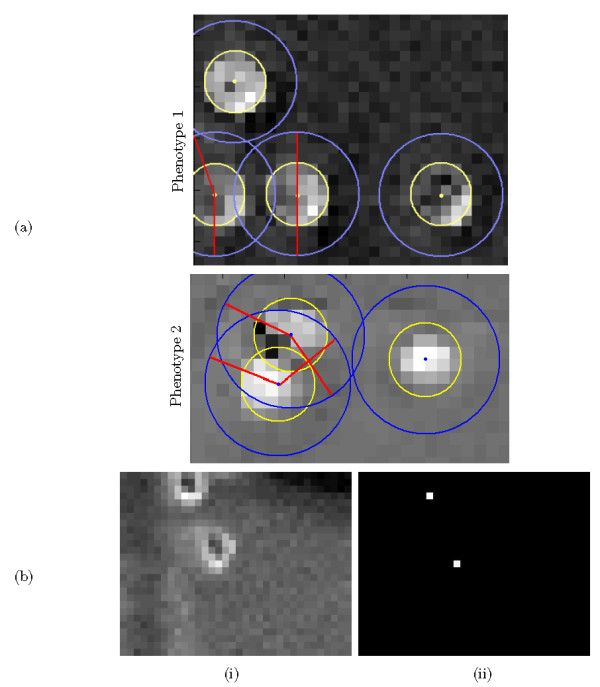
**Cell model**. (a) The proposed cell model (8) assumes a uniform zero-mean background. A cell is modelled by the product of *P*_*cell*_, the cell probability based on cell interior and *P*_*back*_, the penalizing probability based on outer cell ring. Not all pixels surrounding a cell are necessarily background, however we expect at least some fraction as background, and penalize (*P*_*back*_) the mean-square deviation from zero (*P*_*cell*_) of this fraction. The fraction of background pixels which are located between two rings with radii *r *and 2 *r *are illustrated by solid lines for both phenotypes. (b) Cropped well interior: (i) Cropped well interior with no well boundaries. (ii) Applying the cell model to (i).

As we can see in Fig. 2(a), all HSCs *cannot*, in fact, be characterized by model (8) as a well recognizable dark interior and a bright boundary and the previous method in [8] performs poorly to detect them.

Different HSC phenotypes can be characterized as approximately circular objects with high intensity variations against the background, as can be observed in Fig. 2(a). Assuming a uniform background, we designed a general cell detection model in our previous work [9]. This method performed well to detect different HSC phenotypes, however it suffered from discontinuity and spurious detection. Moreover the gray level image was converted to a binary image using Otsu's thresholding at the expense of losing gray level features that could be used to improve cell detection.

Therefore to model the HSCs we propose an improved model, characterized by the following criteria:

1. The cell is round, with some radius.

2. The intensities of cell pixels deviate (both brighter and darker) from those of background pixels.

3. Most of the pixels around the cell are near the background mean.

We propose a revised probabilistic cell model as the product of two probabilistic terms *P*_*cell *_and *P*_*back *_which are associated with the cell brightness and the surrounding background respectively:

(8) $$P(z_{k}^{m}|I_{k})=P_{cell}(z_{k}^{m}|I_{k})\cdot P_{back}(z_{k}^{m}|I_{k})$$

This revised model (8) is similar in sprit to [8], but generalized to different HSC phenotypes investigated in this work. The description and computation of each probabilistic term follows.

### Cell Probability as a Circular Anomaly

A cell is modelled as a circular anomaly, darker or brighter than the uniform background. Let $G(z_{k}^{m},I_{k})$ return a circular set of pixels

(9) G(z,I)={Iij|(x−i)2+(y−j)2≤(r)2}

The cell model assumes the background to have been subtracted, therefore the background mean is zero, and deviations from zero suggest the presence of a cell. Thus we extract the mean-square intensities

(10) $$\bar{G}=\frac{\sum_{g\in G}g^{2}}{\left|G\right|}$$

The cell probability *P*_*cell *_is proposed, based on observations, to be an exponential

(11) $$p_{cell}(z_{k}^{m}|I_{k})=1-exp\{-\bar{G}(z_{k}^{m},I_{k})\}$$

so that *P*_*cell *_has a strong response for cell pixels, and weak (close to zero) for background pixels.

### Penalizing False Candidates

Except for cells which are very tightly packed, most cells will be surrounded by background. Therefore to distinguish a cell center from a point between two adjacent cells, we can test for the presence of background pixels in a ring around the cell, between radii of *r *and 2*r*:

(12) E(z,I)={|Iij||r2≤(x−i)2+(y−j)2≤(2r)2}

Not all of the pixels surrounding a cell are necessarily background, but we do expect at least half of them as background, and penalize the mean-square deviation from zero of this fraction. Let $E^{\frac{1}{2}}$ be the half subset of *E *with intensities closest to the background mean of zero:

(13) $$E^{\frac{1}{2}}=\{e\in E|e<Median(E)\}$$

We then calculate the mean square ${\bar{E}}^{\frac{1}{2}}$

(14) $${\bar{E}}^{\frac{1}{2}}=\frac{\sum_{e\in E^{\frac{1}{2}}}e^{2}}{\left|E^{\frac{1}{2}}\right|}$$

We assume that we have an image sequence with a zero-mean background plus additive noise which, if Gaussian, leads ${\bar{E}}^{\frac{1}{2}}$ to be χ^2 ^when $E^{\frac{1}{2}}$ contains mostly background, so the cell/background hypothesis test can be approximated by a simple exponential:

(15) $$P_{back}(z_{k}^{m}|I_{k})=exp\{-{\bar{E}}^{\frac{1}{2}}(z_{k}^{m}|I_{k})\}$$

### Locating The Cell Centers

To locate the cell centers, we compute the probability map $P(z_{k}^{m}|I_{k})$by applying the cell model in (8) to image frame *I*_*k*_, and then find the cell centers as the thresholded local maxima in $P(z_{k}^{m}|I_{k})$.

**Figure 3:**
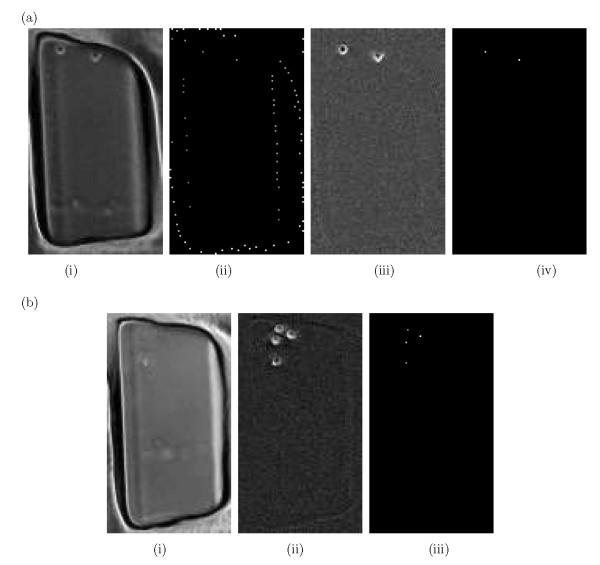
**Background estimation and cell detection**. (a) The proposed cell model: (i) Coarsely cropped well in which well boundaries are visible. (ii) Applying the cell model to (i). In contrast with the perfect result obtained by applying the cell model to a cropped well interior with no boundaries, here the cell model performs very poorly where the cropped well contains visible well boundaries. (iii) Corrected well image after point-wise background subtraction. (iv) Applying the cell model to (iii). (b) The process of the proposed cell model and background estimation method: (i) Point-wise estimated background (${\hat{B}}^{0}$). (ii) Subtracting out the estimated background image obtained in (i) from the original image. (iii) Located cell centers applying cell model *P *in (8).

The threshold is computed analogously to [8], in which the threshold is varied and selected to minimize the sum of missed detections and false alarms. For the proposed cell metric (8), a threshold of 0.25 was found to be very effective, giving a detection rate over 95%, and a false alarm rate of approximately 6%. Fig. 2(b) shows the application of the cell model to a cropped well interior with no boundaries (Fig. 2(b-i)) and a coarsely cropped well before and after background correction is depicted in Figs. 3(a-i) and 3(a-iii) respectively. As it can be observed, the cell model performs very poorly (Fig. 3(a-ii)) where the cropped well contains visible well boundaries, however the cell model performs perfectly where it is applied to a cropped well interior with no boundaries (Fig. 2(b-ii)) or a background corrected coarsely cropped well (Fig. 3(a-iv)).

## Results

**Figure 4:**
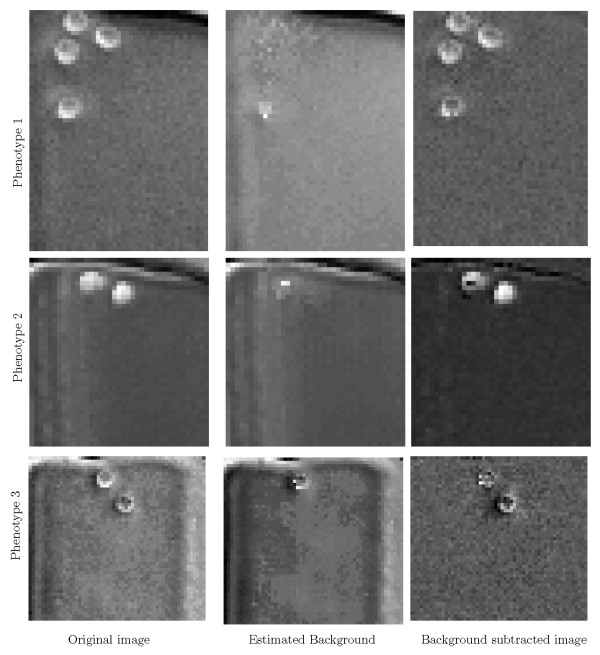
**Estimated background images for three HSC phenotypes are obtained by applying the point-wise method**. As it can be observed, well boundaries and the stationary background pixels are well estimated.

We have applied the proposed cell model and background estimation method to different sequences of phase contrast HSC images. The entire proposed method, consisting of point-wise background estimation for cell detection, and cell detection based on a probabilistic model is applied to a whole, imperfectly cropped well as depicted in Fig. 3(b). Point-wise background removal is illustrated in Fig. 3(b-ii) while cell-center detection is shown in Fig. 3(b-iii). Fig. 4 shows the estimated background and the corrected image frames for different HSC phenotypes under consideration applying the point-wise background estimation/subtraction.

**Figure 5:**
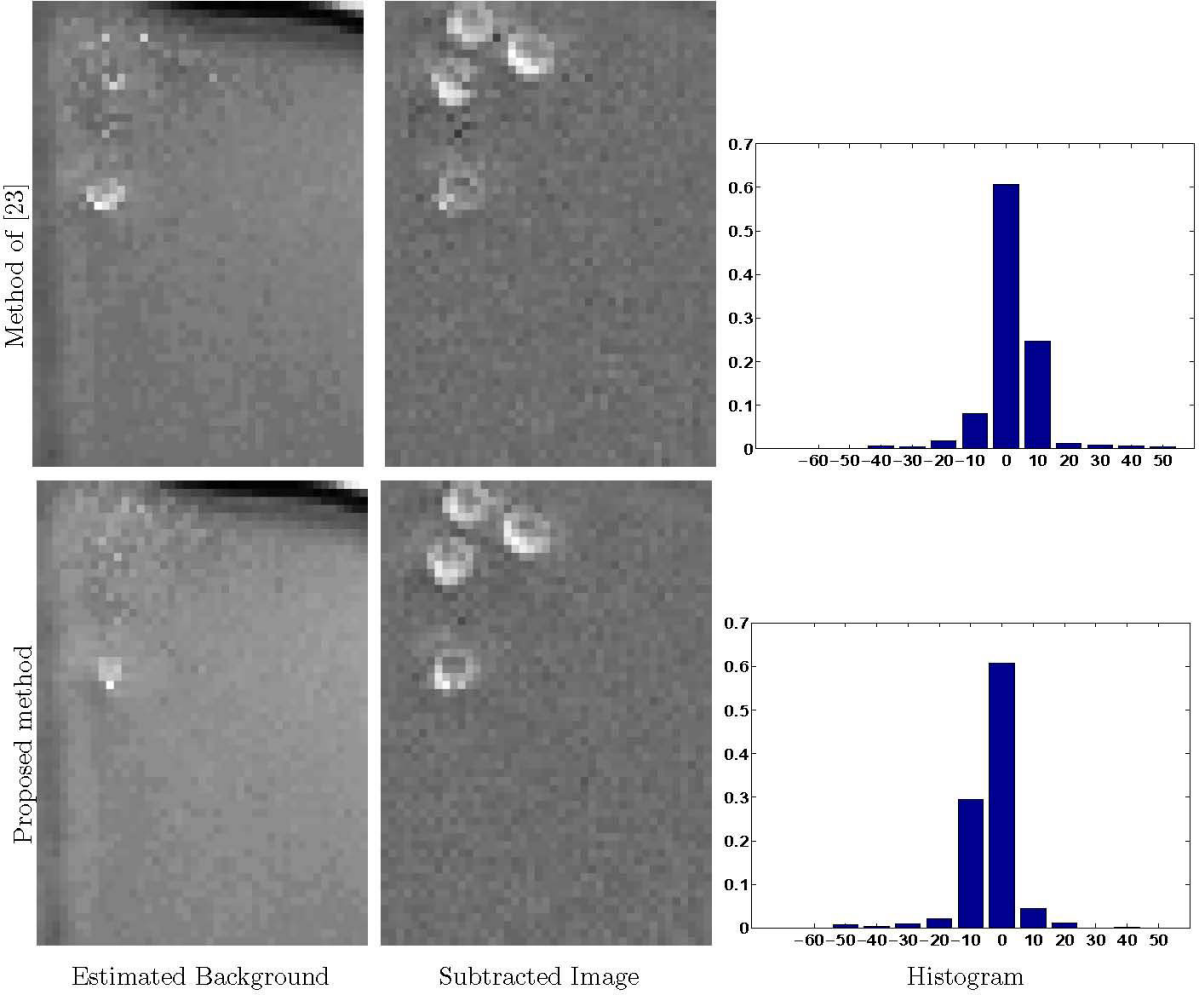
**Estimated background, corrected images, and normalized histograms of top left part of estimated background based on applying **[23]** and the point-wise method ${\hat{B}}^{0}$ for phenotype 1**. The presence of quasi-stationary cells in the mid-left of the image sequence causes [23] to interpret the cells as belonging to the background, leading to a cell-background contrast loss in such locations after background subtraction, as we can see in the subtracted image, whereas our proposed point-wise method has quasi-stationary cells only barely appearing in the estimated background. There is a significant number of pixels with intensities higher than the background in the top normalized histogram, showing that some cell boundary pixels are interpreted as background by [23] increasing the misdetection rate.

The results obtained by the proposed point-wise background estimation method is compared with other methods as follows. First, the proposed method by *Heikkila and Pietikainenin *[23], a recent background modelling method with very promising results, is implemented. To train the background model using LBP (Local Binary Pattern) texture operator we use the same number of frames as we used to estimate the background in our proposed method. Fig. 5 compares the estimated background images of our proposed method with that of *Heikkila and Pietikainenin*. As can be observed, where cells have slow dynamics, cell boundary pixels are apparent in the estimated background images using LBP, leading to significant degradation of the bright cell boundaries, in contrast with our proposed method that precisely estimates background and generates a smooth, uniform background. As can be observed in the normalized histograms, most of the pixels have background of zero, however there is significant number of pixels with intensities higher than background in the top histogram in comparison with the bottom one confirming that some cell boundary pixels are interpreted as background in the estimated background image by [23] which leads to the loss of cell-background contrast and in turn increases the misdetection (missed cells and false alarms) rate.

**Figure 6:**
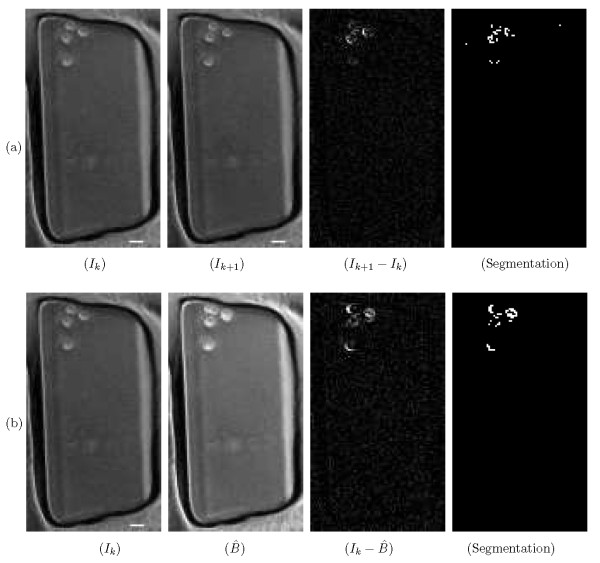
**Other methods of segmentation and background estimation**. (a) Frame difference segmentation: The segmentation of foreground cells based on frame differences. It can be observed that frame-difference segmentation does not yield a satisfactory segmentation result. Segmented cells end up as scattered pixels without any recognizable shape. (b) Morphological background estimation/subtraction: Estimated background and segmentation using morphological operators. Cells are not meaningfully segmented in the foreground that is obtained by applying morphological background estimation/subtraction of [11]. Scale bar is 20 *μm*.

Two additional approaches, a frame-difference segmentation method and a morphological averaging background estimation method in [11], are implemented. Depicted in Fig. 6(a) and 6(b), we can observe that neither frame-difference segmentation nor morphological averaging background estimation provides a satisfactory result. Segmented cells lose their form and become scattered pixels.

**Figure 7:**
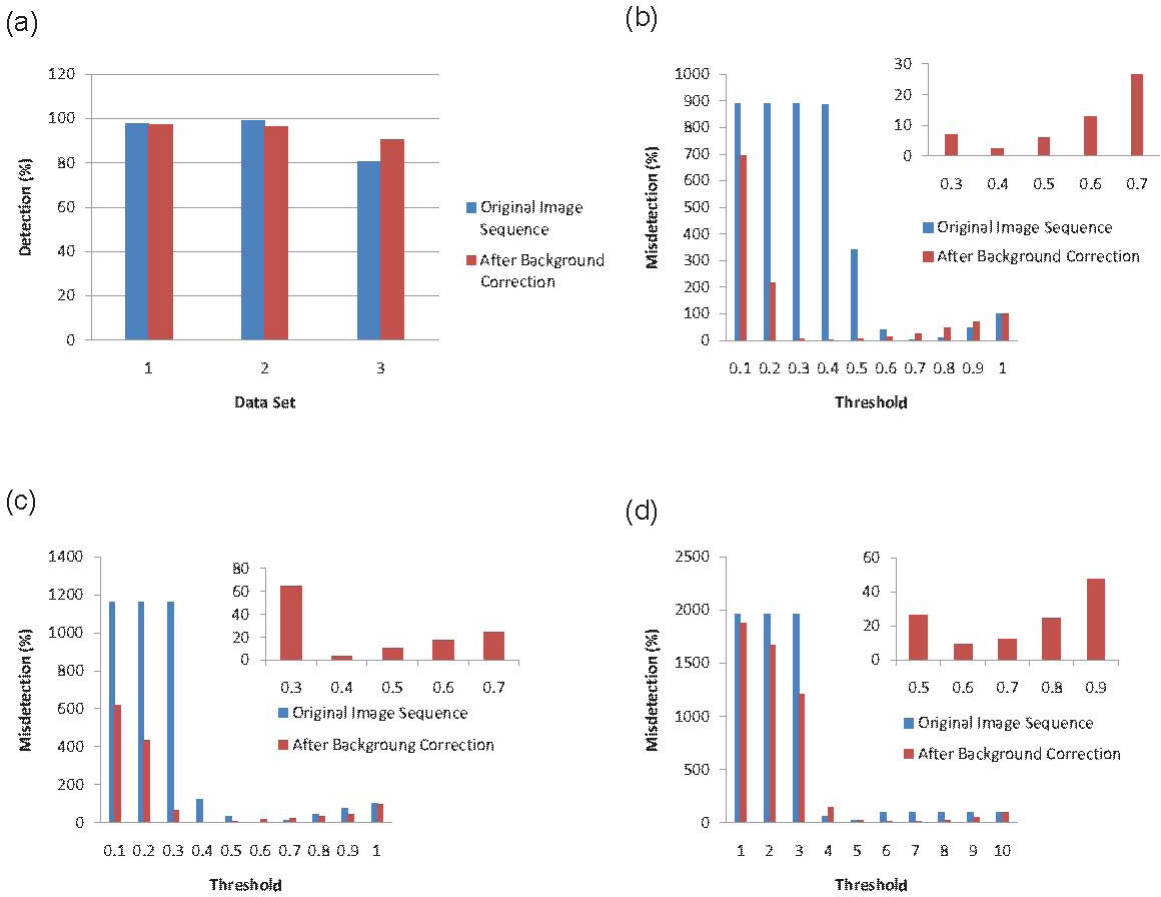
**Cell detection performance**. (a) Detection performance applying the proposed cell model to three different HSC phenotypes. (b), (c), and (d) Misdetection rate as a function of threshold value for phenotypes 1, 2, and 3 respectively.

Background correction is essential for imperfect cropped wells as well as poor contrast image sequences. However cell model can be directly applied to the image sequences of different pheno-types in which well interior is perfectly cropped and no well boundaries is visible. Fig. 7 shows the cell detection results obtained by applying the proposed cell model to the image sequences of cropped well interior with no well boundaries for different phenotypes before and after point-wise background correction. The detection rate for different cell phenotypes is depicted in Fig. 7(a). As it can be observed, even for the worst case (very poor contrast images), the detection rate was over 90% while it reached 98% for phenotypes 1 and 2. The proposed cell model works well with all cell phenotypes while the cell detection improved significantly (about 10%) for poor contrast images using point-wise background correction. The detection rate applying the proposed cell model to phenotypes 1 and 2 is very high (close to 98%) and there is no need to use the point-wise background correction if well structure is not visible in the cropped image sequence. As we can see the detection rate is almost the same before and after point-wise correction for phenotypes 1 and 2.

Figs. 7(b), (c), and 7(d) show the detection rate as a function of the chosen threshold for phenotypes 1, 2, and 3 respectively. As it can be seen, before background correction, the misdetection rate which includes both missed cells and false alarms, is consistently very high for *τ *≤ 0.5 where *τ *is the threshold by which the detected local maxima are filtered. However, the performance of the proposed cell model on background corrected (point-wise) sequences is more robust such that the misdetection rate drops fast and stables below 10% for 0.3 ≤*τ*≤ 0.6 after background correction.

## Conclusions

Information about the temporal dynamics and spatial distribution of cells is beneficial to study the cell behavior. The traditional method of manual observation and measurement of cells from microscopic images is tedious, expensive, and time consuming. Thus, automated methods for the observation and measurement of cells from microscopic images are in high demand.

In this paper, a mutual algorithm for cell detection and background estimation is presented. The proposed probabilistic cell detection approach, assuming a uniform background, models a cell by the product of the cell probability based on cell interior and the penalizing probability based on outer cell ring. The proposed background estimation method estimates the background over the 3-D sequence to remove the well structure. The proposed method was applied to different HSC image sequences and generated promising results. The purpose of developing this method was analysis of single cells, dividing cells, and low packed cells such that by further outlining the boundary of cells, the cell shape, size, and other features can be extracted over time to study the spatiotemporal cell behavior. Although the proposed method can be modified to potentially detect single cells in tightly packed cell cultures (roughly based on the average cell diameter), the boundary of individual cells can not be discriminated. This might be useful for counting the number of cells, however cannot be used for single cell analysis which is the purpose of this paper.

The proposed method is capable of localizing the specific cell types that have been used in our experiments, however to adapt the method for the other cell types that may not have circular shape, this method must be modified to model the specific cell shape.

Further, the proposed method can potentially be used for detection of different stem cell types; however it has not been tested for detecting cells in stem cell aggregates, in particular for circumstances in which neighboring cells significantly overlap with each other, and where cell morphology may considerably change.

The proposed algorithm was applied to bright field cell images, so in situations that cells are fluorescent (for example by adding calceinAM) or where the user needs to distinguish features across cells such as antibody stained cells, the proposed method must be modified to properly model the cell for the fluorescence range of interest. In such cases, if the color stain would spill outside the cell boundary or partially stain the cell area, the cell detection might drop in accuracy.

## Competing interests

The authors declare that they have no competing interests.

## Authors' contributions

NNK designed and implemented the algorithm and drafted the manuscript. NNK and PF discussed the methods and modified them. PF read, commented on, and edited the manuscript. EJ provided the image sequences and commented on the methods. All authors read and approved the final manuscript.
